# Can Fetuin-A Be a Marker for Insulin Resistance and Poor Glycemic Control in Children with Type 1 Diabetes Mellitus?

**DOI:** 10.4274/jcrpe.4532

**Published:** 2017-12-15

**Authors:** Ülkü Gül Şiraz, Murat Doğan, Nihal Hatipoğlu, Sabahattin Muhtaroğlu, Selim Kurtoğlu

**Affiliations:** 1 Erciyes University Faculty of Medicine, Department of Pediatric Endocrinology, Kayseri, Turkey; 2 Erciyes University Faculty of Medicine, Department of Biochemistry, Kayseri, Turkey

**Keywords:** Non-alcoholic fatty liver disease, fetuin-a, type 1 diabetes mellitus, complication

## Abstract

**Objective:**

Metabolic impairment in type 1 diabetes mellitus (T1DM) with poor glycemic control causes insulin resistance, non-alcoholic fatty liver disease (NAFLD), atherosclerosis, and increased carotid intima-media thickness (CIMT). Fetuin-A has a protective effect in cardiovascular disorders and is increased in hepatosteatosis. We aimed to investigate the reliability of fetuin-A levels in early detection of diabetic complications in children with T1DM and to identify a cut-off value that may show poor metabolic control.

**Methods:**

The study included 80 patients who had T1DM for at least 5 years and who had no chronic complications or an auto-immune disorder. Blood samples were drawn to measure hemoglobin A1c (HbA1c), biochemical parameters, and fetuin-A levels. Anthropometric parameters were also measured. Percent body fat was calculated. Hepatosteatosis and CIMT were assessed by sonography.

**Results:**

Mean age of the patients was 13.5 years. Grade 1 hepatosteatosis was detected in 10%. Patients were stratified into 2 groups based on presence of NAFLD. Fetuin-A level was increased in patients with NAFLD. We identified a fetuin-A cut-off value (514.28 ng/mL; sensitivity: 47.34; specificity: 96.72) that may predict NAFLD. HbA1c and total cholesterol levels were found to be higher in patients with fetuin-A levels above higher the cut-off value.

**Conclusion:**

Fetuin-A is a reliable parameter in the prediction of complications and poor glycemic control in patients with T1DM.

**What is already known on this topic?**

Hyperlipidemia and hyperglycemia lead to an increase in fetuin-A production.

**What this study adds?**

Fetuin-A levels could be useful in predicting complications such as hepatosteatosis and atherosclerosis in patients with type 1 diabetes mellitus.

## INTRODUCTION

Type 1 diabetes mellitus (T1DM) is more commonly seen at childhood. Children with T1DM are classically lean or of normal weight at the time of diagnosis. In recent years, in parallel to increased incidence of obesity, increased body weight and development of insulin resistance have become striking findings in patients with T1DM, particularly in those with poor glycemic control (1). Both glucotoxicity and metabolic imbalance caused by poor glycemic control lead to more severe complication at earlier ages, implying a need for a novel and practicable criterion for early diagnosis and monitoring of complications.

Hepatosteatosis is an important complication in children with T1DM as it indicates metabolic imbalance and development of peripheral insulin resistance. Lipoprotein is structurally impaired, and secondary to the hyperglycemia, glucose transporter 2-mediated glucose uptake from the circulation by the liver is increased. This results in increased fatty acid and lipoprotein synthesis, which is implied in the pathogenesis (2,3,4). Such hepatic disorders due to causes other than alcohol are termed as non-alcoholic fatty liver disease (NAFLD). In addition, hyperglycemia can also cause endothelial injury and activation of the coagulation system (5,6). In this regard, carotid intima-media thickness (CIMT) that reflects premature atherosclerotic changes is an important marker (7). Thus, hepatosteatosis and increased CIMT are complications which may herald poor metabolic control and chronic influences in children with T1DM (8).

Fetuin-A (Alpha-2 Heremans Schmid glycoprotein) is a negative acute phase reactant synthesized by the liver. This glycoprotein also causes insulin resistance by enhancing insulin receptor tyrosine kinase activity and insulin receptor auto-phosphorylation (9). The fact that fetuin-A production is increased by hyperlipidemia and hyperglycemia suggests that it can be a valuable parameter for predicting complications of T1DM as well as monitoring poor glycemic control (10).

This study aimed to investigate fetuin-A levels in predicting complications in patients with T1DM and to assess its relationship with clinical, radiological, and biochemical parameters. It also aimed to identify a cut-off value for fetuin-A that may indicate poor metabolic control and to assess its reliability.

## METHODS

The study was approved by the Erciyes University Local Ethics Committee (approval number: 2014/118) and informed consent was obtained from patients or relatives. The study included 80 patients (40 boys and 40 girls, mean age 13.5 years) without any chronic complications or comorbid auto-immune disorders and who had been followed with the diagnosis of T1DM for at least 5 years at the Pediatric Endocrinology Department of Erciyes University, Faculty of Medicine. Patients with alcohol consumption, smokers, those with positive serology for hepatitis, those with an infectious or comorbid systemic disorder, and those on drug therapy were excluded. In all patients, weight (kg), height (cm), waist circumference (cm), and neck circumference were measured by the same observer. Body mass index (BMI) was calculated by using the following formula: BMI = weight (kg) / height2 (m2). Patients with a BMI >95th percentile according to age and gender were considered as obese (11). The waist circumference was measured at the narrowest level between the costal arch and the processus spina iliaca anterior superior at the end of expiration in the patient standing upright (12). The neck circumference was measured at the level of the superior margin of the cricothyroid membrane in the patient standing upright with shoulders relaxed (13). Two blood pressure measurements with 15 minutes interval were performed by the same observer and the mean value was recorded.

Percentage of body fat (BF%) was analyzed by using Tanita BC480MA body composition analyzer. Blood samples were drawn after an 8-hour fast for determination of lipid profile [triglyceride, total cholesterol (TC), low-density lipoprotein (LDL), high-density lipoprotein], hepatic enzymes [alanine aminotransferase (ALT), aspartate aminotransferase (AST)], gamma-glutamyl transferase (GGT), glycated hemoglobin A1c (HbA1c), serological hepatitis markers, systemic infection markers, and complete blood count (CBC). In addition, microalbumin: creatinine ratio was measured in spot urine samples of first morning void. Microalbumin: creatinine ratio of 0-30 µg/mg was defined as normal, while 30-300 µg/mg as micro-albuminuria and >300 µg/mg as proteinuria. Biochemical parameters were determined using Abbot Architect C8000 kits, while CBC was performed by using Siemens Advia 2120i hematology system.

Fundus examination and electromyography were performed in all patients. Children with diabetic micro-vascular complications (microalbuminuria-neuropathy-retinopathy) or comorbid auto-immune disorders were excluded.

Sonography was performed by the same observer in all patients. Internal carotid artery and the segment 1-2 cm proximal to the main carotid artery were screened and CIMT measurement was performed. To assess NAFLD, hepatic sonography was performed and rated as grade 1, 2, and 3 according to degree of hepatic echogenicity and appearance of right hemi-diaphragm and intra-hepatic vessels.

Blood samples (5 mL) were drawn into anticoagulant-free tubes for fetuin-A analysis. After allowing clotting over 20-30 minutes at room temperature, the samples were centrifuged at 5000 rpm for 10 minutes at 4±2 °C in a refrigerated centrifuge. The sera were stored at -80 °C until time of assay. Fetuin-A was measured using the enzyme-linked immunosorbent assay technique by taking the dilution factor into account. The detection limit was 0.35 ng/mL. Intra-assay and inter-assay coefficients of variation were 3.5% and 5.5%, respectively (14).

### Statistical Analysis

The data were analyzed by using IBM SPSS Statistics version 22.0. Descriptive statistics were presented as count (n), percent (%), mean ± standard deviation, and median (minimum-maximum). In numeric variables, normal distribution was tested by Shapiro-Wilk normality test and Q-Q graphics. Comparisons between groups were performed by using the non-parametrical Mann-Whitney U test, while prevalence comparisons were performed by using chi-square test. A p-value <0.05 was considered as statistically significant. Receiver operating characteristics (ROC) analysis was used to determine cut-off value for fetuin-A and its reliability. The Youden index (represents the maximum of sensitivity + specificity -1 for all cut points in the ROC curve) was used to determine the optimum cut-off value of fetuin-A for detection of hepatosteatosis.

## RESULTS

The study included 80 patients with T1DM (40 boys and 40 girls). Duration of DM ranged from 5 to 16 years. On sonography, grade 1 hepatosteatosis was detected in 8 patients (10%).

The patients were stratified into two groups based on presence or absence of hepatosteatosis, as NAFLD (+) and NAFLD (-). The groups were compared regarding age, gender, duration of DM, BMI, BF%, lean body mass (LBM), right and left CIMT, fetuin-A level, HbA1c level, lipid profile, and results of hepatic function tests. Mean blood pressure was assessed according to age- and sex-adjusted percentile curves. No patient had hypertension based on this assessment.

Mean age was 14 years (9-17 years) in NAFLD (+) patients and 13.5 years (6-18 years) in NAFLD (-) patients. There were 5 boys and 3 girls in the NAFLD (+) group, 35 boys and 37 girls in the NAFLD (-) group. There was no significant difference in age and duration of DM between the groups (p=0.37 and p=0.089; Table 1).

When groups were compared, BF% and right-left CIMT were found to be significantly higher in the NAFLD (+) group than in the NAFLD (-) group (p=0.037, p=0.003 and p=0.014, respectively). No significant difference was detected in anthropometric measurements, BMI, and LBM between the two groups.

**Figure 1:**
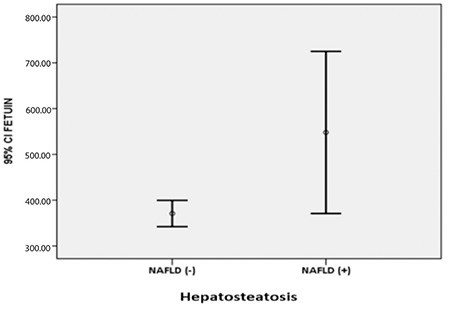
Relationship between fetuin-A and non-alcoholic fatty liver disease NAFLD: non-alcoholic fatty liver disease, CI: confidence interval

When biochemical parameters were compared between groups, fetuin-A and ALT levels were found to be significantly higher in the NAFLD (+) group (p=0.024 and p=0.072, respectively; Table 2, Figure 1). AST: ALT ratio was significantly lower in the NAFLD (+) group. There was no significant difference in HbA1c, GGT, and lipid levels between the two groups.

The ROC analysis was used to assess usefulness of fetuin-A as a marker for hepatosteatosis development. In the ROC analysis, area under curve was estimated to be 0.672 [95% confidence interval (CI): 0.558-0.773; p=0.022], indicating that fetuin-A is a reliable parameter. Again, in the ROC analysis, cut-off value for presence or absence of hepatosteatosis was calculated as 514.28 ng/mL (sensitivity: 47.34, 95% CI: 24.5-71.1 and specificity: 96.72, 95% CI: 86.6.99.5). Youden index was calculated as 0.51.

We also assessed the reliability of fetuin-A cut-off value as a marker for poor metabolic control. The statistical relationship between fetuin-A level above cut-off value and NAFLD positivity was assessed by chi-square test, revealing a significant difference (x2: 20.476, p<0.001).

**Figure 2:**
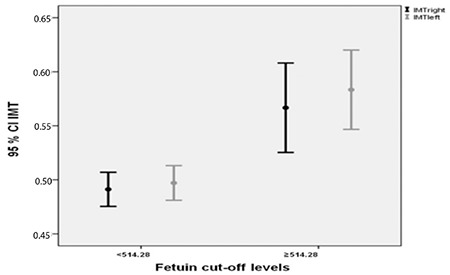
Relationship between fetuin-A and carotid intima-media thickness CIMT: carotid intima-media thickness, IMT-right: intima-media thickness-right, IMT-left: intima-media thickness-left

When clinical findings between groups were analyzed by stratifying patients according to cut-off value, it was found that patients with a fetuin-A level above cut-off value had higher BF% value and increased right and left CIMT (p=0.001, p=0.001, and p<0.001, respectively; Table 3; Figure 2). However, there was no significant difference in age, duration of DM, BMI, LBM, waist circumference (WC), and neck circumference between the two groups.

When biochemical parameters were analyzed by stratifying patients according to cut-off value, it was found that there were significant differences in HbA1c, GGT, and TC levels between patients with a fetuin-A level below and above cut-off value (p=0.03, p=0.025, p=0.027, respectively), but there was no significant difference in ALT level nor in AST: ALT ratio (Table 4).

HbA1c, AST, GGT, BF%, and WC were detected to positively correlate with fetuin-A (r=0.370, p=0.001 – r=0.274, p=0.015 - r=0.419, p<0.001 - r=0.485, p<0.001 - r=0.286, p=0.010, respectively).

## DISCUSSION

The prediction of complications which determine mortality and morbidity in T1DM before onset of clinical findings, at a time when they are reversible, will increase success in the follow-up (15). In the present study, fetuin-A levels were investigated as a parameter to determine risk prediction.

In the literature, there is limited number of studies on association of NAFLD and poorly controlled T1DM. In a study from Egypt, NAFLD was detected in 4.5% of 692 children with T1DM (16). In a study on 202 patients with T1DM, Targher et al (8) detected hepatosteatosis in 44.4% of patients and reported higher HbA1c level, longer DM duration, and higher nephropathy rates in these patients. In that study, it was seen that mean age was higher in diabetic patients with NAFLD than in those without NAFLD (47±12 years vs. 37±12 years). In our study, there was NAFLD in 10% of 80 patients with T1DM; however, no significant difference was detected between patients regarding duration of DM. In addition, no micro- or macro- vascular complications were detected in the patients. Lack of significant difference in age and duration of DM and absence of complications may be attributed to selection of younger patients.

The most valuable marker of hepatic injury is elevated ALT levels and low AST: ALT ratio (17,18). In our study, it was found that ALT levels were higher, while AST: ALT ratio was significantly lower in patients with NAFLD.

In a study from a pediatric diabetes centre in Germany, 93 children and adolescents with T1DM were investigated for NAFLD using ultrasound, biochemical findings, and liver stiffness measurements (FibroScan® and acoustic radiation force imaging). Completely normal results were obtained in 88.1% of these patients, 1.1% fulfilled the criteria as potential NAFLD patients, and 10.8% showed some mild abnormality in at least one category (19). We obtained similar results in this present study, using a different technique to evaluate NAFLD.

The CIMT is used as an early marker of subclinical atherosclerosis, and it is reported that CIMT is higher in diabetic patients than in the normal population (20,21). Gökşen et al (20) conducted a study on 55 children with T1DM and 30 healthy children and found that CIMT values were significantly higher in diabetic patients when compared to healthy controls. In our study, there was no control group; however, it was found that bilateral CIMT value and BF% were higher in diabetic patients with NAFLD despite comparable BMI values, as well as comparable duration of DM and HbA1c levels.

Although it is unclear which factors regulate fetuin-A production, it was reported that free fat acids (FFAs) and hyperglycemia increase fetuin-A expression and induce insulin resistance (22). Ix et al (23) conducted a study to investigate the relationship between metabolic syndrome and fetuin-A on 711 patients with coronary artery disease having non-diabetic metabolic syndrome and found that the increase in fetuin-A level was associated to each component of metabolic syndrome. Authors suggested that this was due to increased lipolysis as a result of inhibition of tyrosine kinase receptor by fetuin-A, increased release of FFAs from adipose tissue, and increased production of apolipoprotein B containing very LDL (24).

In a study on rats with induced T1DM, it was demonstrated that secondary insulin resistance developed immediately after deprivation of insulin reserve and that this was associated with fetuin-A levels (24). Although the number of clinical trials on this issue is limited, in the study by Gheissari et al (25), a significant decrease was observed in fetuin-A level after initiation of valsartan in T1DM patients with nephropathy. In another study in which patients with T1DM (n=62) were compared with healthy controls, fetuin-A level and CIMT were found to be higher in the patient group (26). These findings have raised the question as to whether increased fetuin-A levels can predict development of findings of metabolic syndrome, such as insulin resistance, hyperlipidemia, NAFLD and increased CIMT values are added over time, in T1DM patients with poor glycemic control. In our study, both fetuin-A and CIMT values were found to be higher in patients with NAFLD. In the second stage of our study, using ROC analysis, a cut-off value of fetuin-A for NAFLD risk was calculated to be 514.28 ng/mL. The sensitivity and specificity were 47.34 (95% CI: 24.5-71) and 96.72 (95% CI: 86.6-99.5), respectively. When patients were stratified according to this cut-off value, it was seen that patients with fetuin-A levels above the cut-off value had poorer glycemic control and higher TC levels. However, there was no significant difference in ALT level and AST: ALT ratio between the two groups. These findings may indicate that fetuin-A levels may be a marker for hyperlipidemia, metabolic syndrome, and poor glycemic control before onset of hepatic injury in children with T1DM. Again, bilateral IMT values were significantly higher in patients with fetuin-A levels above the cut-off value; this finding supports the hypothesis that fetuin-A is a marker of early atherosclerotic changes.

### Study Limitations

We acknowledge that the small number of T1DM patients diagnosed as hepatosteatosis in our study is a limitation. Also, our reason for using sonography instead of fine-needle biopsy, which is the accepted gold standard in the diagnosis of hepatosteatosis, was due to its being a non-invasive method.

## CONCLUSION

In conclusion, our findings indicate that increased CIMT values and NAFLD can be accepted as markers of poor metabolic control in the follow-up of diabetic patients before detectible diabetic complications develop. The results also suggest that patients at risk can be identified by measuring fetuin-A level alone before onset of clinical and/or laboratory findings of chronic complications. Further comprehensive studies are needed in this area.

## Figures and Tables

**Table 1:**
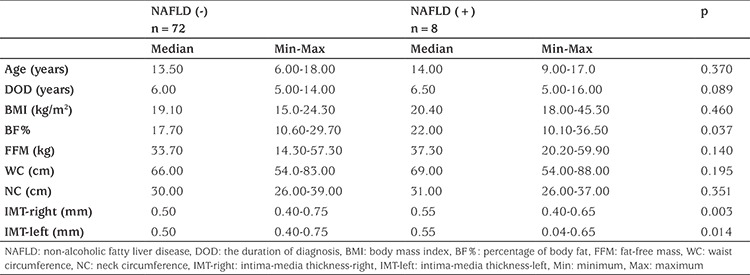
Assessment of patients according to hepatosteatosis

**Table 2:**
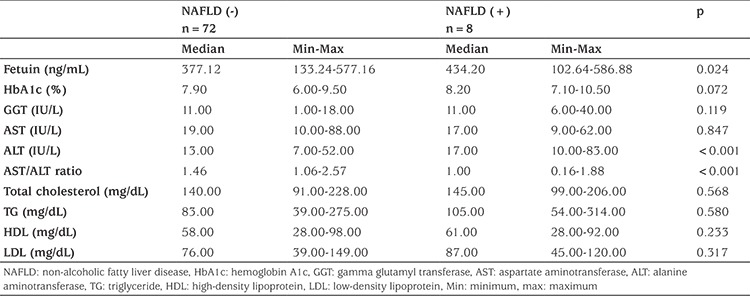
Biochemical parameters according to non-alcoholic fatty liver disease

**Table 3:**
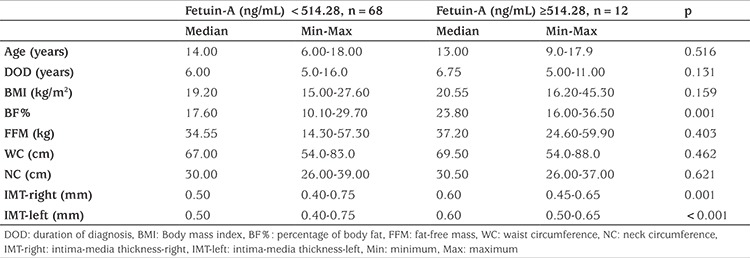
Anthropometric measurements according to fetuin-A cut-off levels

**Table 4:**
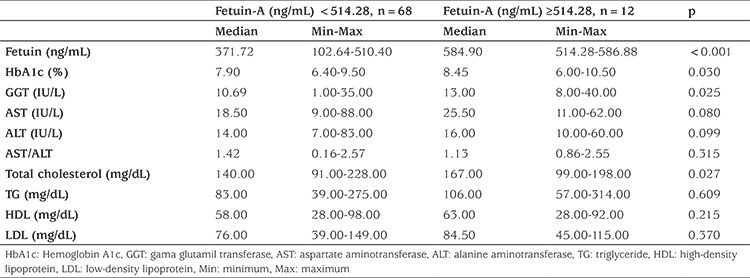
Biochemical parameters according to fetuin-A cut-off levels
